# Seroprevalence of *Coxiella burnetii* in patients presenting with acute febrile illness at Marigat District Hospital, Baringo County, Kenya

**DOI:** 10.1002/vms3.493

**Published:** 2021-05-06

**Authors:** Allan P. Lemtudo, Beth K. Mutai, Lizzy Mwamburi, John N. Waitumbi

**Affiliations:** ^1^ United States Army Medical Research Directorate‐Africa/Kenya Walter Reed Army Institute of Research/Kenya Medical Research Institute Kisumu Kenya; ^2^ Department of Biological Sciences School of Science University of Eldoret Eldoret Kenya

**Keywords:** *Coxiella burnetti*, Q fever, zoonosis

## Abstract

Q fever is not routinely diagnosed in Kenyan hospitals. This study reports on Q fever in patients presenting at Marigat District Hospital, Kenya, with febrile illness. ELISA was used to detect *Coxiella burnetii* phase antigens. Of 406 patients, 45 (11.1%) were judged to have acute disease (phase II IgM or IgG > phase I IgG), 2 (0.5%) were chronic (phase I IgG titer >800 or phase I IgG > phase II IgG), while 26 (6.4%) had previous exposure (phase I IgG titer <800). Age (6–10 years, *p* = 0.002) and contact with goats (*p* = 0.014) were significant risk factors. Compared to immunofluorescence antibody test, the sensitivity and specificity for phase I IgG were 84% and 98%, respectfully, 46% and 100% for phase II IgG and 35% and 89% for phase II IgM. It is concluded that the low sensitivity of phase II ELISA underestimated the true burden of acute Q fever in the study population.

## Graphical abstract

The study assessed immune responses to *C. burnetii* phases 1 and II antigens and inferred the presence of acute and chronic Q‐fever in a pastoral community presenting with febrile illness at Marigat District Hospital, Kenya. Risk factors to the disease included gender, age, season and animal contact.

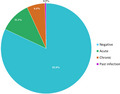

## 1 INTRODUCTION

*Coxiella burnetii* is an obligate intracellular Gram‐negative coccobacillus that causes Q fever and is infectious to both humans and animals. The causative agent was initially unknown; hence, ‘Query’ fever and was later named Q fever. It is mainly an airborne zoonosis with public health concern throughout the world. The disease was first described in 1935 in Queensland, Australia, during an outbreak of a febrile illness of unknown origin among abattoir workers (Angelakis & Raoult, 2010; Honarmand, 2012). Similarly, all the seven cases reported in Taiwan had history of animal contact (Ko et al., 1997). With infections carrying the largest burden of FUO (>70%; Baymakova et al., 2016), *C*. *burnetii* has been implicated as one of the leading causes of undifferentiated fevers (Baymakova et al., 2019; Krumova et al., 2019) and thrombocytosis (Lemos et al., 2011).

The bacterium exists as phase I and phase II antigenic forms that can be differentiated by their lipopolysaccharides composition (Porter et al., 2011). Antibody response to these phase variants is utilized to differentially sero‐diagnose acute and chronic forms of the disease. In acute form, the antibody levels to phase II antigen predominate. The reverse is true for chronic Q fever. IFA titers to phase I antigen ≥ 1:800 are considered diagnostic for chronic Q fever (Anderson et al., 2013; Seitz, 2014).

Infected ruminants are the major reservoirs of the infection, but the bacteria can also infect a wide variety of animals, birds and arthropods. Human infections occur mainly by inhalation of aerosolized bacterial spores excreted from infected animal reservoirs or by contact of contaminated animal products and more importantly, abortion materials such as placentas which harbor huge quantities of the bacteria (Angelakis & Raoult, 2010; Porter et al., 2011).

Q fever is listed among the emerging infectious diseases by the World Health Organization, Food and Agricultural Organization, Centre for Disease Control and prevention, and European Food Safety Authority. Because *C*. *burnetii* is very infectious (one bacterium is enough to cause the disease) and can be aerosolized, it is considered a potential biological weapon (Madariaga et al., 2003; McQuiston & Childs, 2002). Q fever has been reported in most countries worldwide with the exception of New Zealand, making it a public health concern throughout the world (Maurin & Raoult, 1999). The disease has increasingly gained attention since one of the largest outbreaks in Netherlands in 2007–2009, in which over 3,500 human cases were reported (Roest et al., 2011).

In Kenya, serological evidence of Q fever in patients with febrile illnesses was first reported in Nakuru in 1955 (Craddock & Gear, 1955). In a sero‐survey conducted in Asembo, western Kenya, a seroprevalence of 30.9% in patients with clinical signs suggestive of Q fever (acute lower respiratory illness) was reported (Knobel et al., 2013). In contrast, in the neighbouring northern Tanzania, a sero‐prevalence of 5.0% was reported in hospitalized malaria free febrile patients who were admitted in two hospitals between September 2007 and August 2008 (Prabhu et al., 2011). Recently, a study in western Kenya conducted on febrile children aged 1–12 years revealed Q fever prevalence of 12.9% (Maina et al., 2016).

Q fever prevalence varies in different geographical regions of the world, as well as in population of differing socioeconomic characteristics (Hartzell et al., 2008; Wardrop et al., 2016). In Kenyan hospitals, Q fever is not routinely diagnosed and subsequently, it is not treated despite the availability of affordable antibiotics such as doxycycline. Furthermore, in a small proportion of the patients, the disease progresses to chronic stage characterized by severe endocarditis. Case fatality rates are 1%–2% in acute cases and 65% in chronic cases (Angelakis & Raoult, 2010; Porter et al., 2011). Q fever was implicated as the cause of AFI outbreak that claimed the lives of six people in East Pokot Subcounty of Baringo in 2014 (Zoonotic Disease Unit, 2014), illustrating clearly the potential of Q fever to cause mortality and morbidity amongst pastoral communities who keep huge herds of livestock.

This study used ELISA to determine the seroprevalence of acute, chronic, past Q fever and the associated risk factors in febrile patients presenting at Marigat District Hospital. The utility of ELISA was evaluated against IFA, the gold standard test for Q fever.

## 2 MATERIALS AND METHODS

### 2.1 Ethical approval

The study protocol was reviewed and approved by Kenya Medical Research Institute's Scientific and Ethical Research Unit (KEMRI SERU #1282) and the Walter Reed Army Research Institute of Human Subject Protection Branch (WRAIR #1402).

### 2.2 Study site and design

Blood samples were collected from Marigat Sub County Hospital, in Baringo County of Kenya. This hospital was chosen because the communities who access health care at the hospital are pastoralists and keep large herds of cattle, sheep and goats. The study used archived serum samples collected between December 2009 and March 2013.

### 2.3 Sample size

A sample size of 406 samples was used to estimate prevalence. A subset of 93 samples were used to compare the performance of ELISA compared to IFA.

### 2.4 ELISA for detection of phase specific antibodies

ELISA kits (SERION ELISA, Classic Institut Virion\Serion GmbH, Würzburg Germany) were used to detect the presence of IgG to phase I and II and IgM to phase II antigens using the manufacturer's test procedure. As stated in the test kit, the sensitivity of phase II IgM test is 94.2% and a specificity 99.3%, while the sensitivity of phase II IgG test is 92.5% and a specificity >99%. The phase I IgA/IgG sensitivity is 94.2% and a specificity of 96.2%. Briefly, before running the tests, samples were diluted to 1:100 for Phase II IgM and Phase I IgG and 1:500 for phase II IgG. The diluent comprised ready‐to‐use dilution buffer made of a protein containing phosphate buffer and tween 20. For phase II IgM assays, serum was first pretreated with the diluting buffer containing rheumatoid absorbent factor (SERION Rf‐Absorbent), then incubated at room temperature (20°C to 23°C) for 15 min or at 4°C overnight. Each 100 µl of prepared serum and ready‐to‐use positive, negative and standard control sera were added as appropriate to respective ELISA plate, sparing one well for substrate blank and then incubated in a moist chamber maintained at 37°C for 60 min. The plates were then washed four times in automated microplate washer (BioTek Instruments, Inc.) in which each wash was done by dispensing 300 µl of wash buffer (sodium chloride solution with Tween 20 and 30 mM Tris/HCl, pH 7.4 containing <0.1% sodium azide) as a preservative. This was followed by aspiration and then dried by manually tapping the plate on paper towels; 100 µl of ready‐to‐use conjugate of anti‐human IgG or IgM polyclonal antibody, conjugated to alkaline phosphatase, stabilized with protein stabilization solution and preservative (<0.1% methylisothiazolone and <0.1% bromnitrodioxane) was added to respective wells except for the substrate blank and incubated for 30 min as described above.

Washing was repeated as described above and 100 µl of ready‐to‐use substrate, p‐nitrophenylphosphate added to all the wells including the substrate blank and incubated for 30 min in a humid chamber. The enzyme reaction was stopped by addition of 100 µl stop solution (0.1 N sodium hydroxide 40 mM EDTA). The optical density (OD) was read at 405 nm on a microplate reader (VersaMax™, molecular devices) preset to shake the plate for 5 s before reading the OD. Data were accepted when validity criteria per run was met, in which a substrate blank must have an OD of <0.25, a positive control and negative control must meet the lot specific OD ranges set by the manufacturer and the variation OD of the cut‐off serum or standard serum must not have exceeded 20% in the duplicate wells. For quality control and tracking assay variability over time, the OD values of the positive and negative controls were monitored by plotting them on Levy Jennings chart. Data were analysed as recommended by the ELISA kit manufacturer whereby the IgG phase I and IgM phase II are reported as either present or absent and quantitatively for IgG phase II antigens.

IgG phase I and IgM phase II were considered positive whenever the measured OD was more than 10% above the extinction of the cut‐off control. Equivocal results were treated as negative. IgG phase I and IgM phase II extinctions were expressed in OD values. IgG phase II extinctions were expressed in U/ml using a logistic‐log‐model calculation and were defined as positive when the titer was ≥30 U/ml. Each IgG phase II run was adjusted to fit manufacturer established lot‐specific four parameter logistic standard curve that was analysed by use of SERION activity V12 Evaluation of the test excel macros tool provided by the manufacturer. Seropositive samples were staged into acute if they had phase II IgM with or without IgG, or had IgG titers to phase II > IgG phase I, even in the absence of phase II IgM. Chronic infection comprised IgG to phase I with titer ≥800 or IgG to phase I > IgG to phase II. Past infection comprised IgG to phase I titers <800.

### 2.5 Indirect immunofluorescence assay

Indirect immunofluorescence assay using the *C*. *burnetii* I+II kit (Vircell microbiologist Granada) was performed according to the manufacturer's instructions. Briefly, the kit contained 10 slides with 10 pairs of wells per slide. Each pair of wells contained formaldehyde inactivated and acetone fixed phases I and II antigens of *C*. *burnetii* Nine Mile strain (ATCC616‐VR) affixed on the respective well pair and labeled as I and II. For IgG determination, 5 μl of sera diluted to 1/64 in phosphate buffered saline (PBS) were added carefully to both pair of wells. The slides were then washed carefully by a gentle stream of PBS, avoiding directing the water jet on the wells, immersed in PBS for 10 min and briefly dip washed in distilled water; 5 μl of ready‐to‐use anti‐human IgG‐fluorescein isothiocyanate (FITC) conjugate was added to each well. The slides were then incubated for 30 min in the dark to avoid quenching of FITC fluorescence, washed as stated above and air dried. All the incubations were performed at 37°C in a humid chamber. For IgM testing, sera were first diluted 1:1 in PBS and treated shortly with an anti‐human IgG sorbent to a final dilution of 1/24 using the sorbent diluent provided in the kit. Then, 5 µl of the prepared sera were added to the well spots. The slides were then incubated for 90 min. Slides were further processed as above but using anti‐human IgM‐FITC conjugates. Ready‐to‐use positive and negative control sera from the kit were included in each slide. After incubation with FITC labeled secondary antibody, the slides were washed and air dried. Finally, a small drop of mounting medium provided in the kit was added to each well and covered with a cover slip. Examination of the slides was performed using an Olympus BX41 fluorescent microscope (Olympus Corporation) at a magnification of 400X.

### 2.6 Data management and analysis

The analysed data were derived from *C*. *burnetii* serological OD values and IFA fluorescence. Sociodemographic and risk information were obtained from epidemiological data provided by the patients. Statistical analysis was performed using STATA software version 12.0 (Stata Corp LP). Descriptive data were expressed as percentages and frequencies. Differences between groups were compared using Chi‐square or Fisher's exact as appropriate. Univariate logistic regression was used to test for associations between sero‐reactivity and potential risk factors. Odds ratios (OR) were calculated and used to assess the strength of association between the dependent and independent variables. For sensitivity, specificity and the positive and negative predictive values calculation, IFA values were used as reference (Buendía et al., 2001). All statistical tests were performed at 5% significance level and corresponding 95% confidence intervals.

## 3 RESULTS

### 3.1 Study participants demographics

A total of 406 serum samples were available for evaluation. The demographic characteristics of the individuals who contributed the samples are shown in Table 1. The mean age was 13.9 + 12.7 years, with majority (67%) being children below 10 years. Females constituted 52.6% of the study population.

**TABLE 1:** Prevalence of *Coxiella* *burnetii* antibodies in febrile patients attending Marigat Sub County Hospital, Kenya

Variable	Level	Tested (*n*)	Positive (*n*)	Seroprevalence (%)	*p* value
Sex	Male	192	42	21.9	0.056
	Female	213	31	14.6	
Age (years)	≤5	104	23	22.1	0.002
	6–12	147	35	23.8	
	13–19	66	10	15.2	
	20–35	61	2	3.3	
	≥35	27	3	11.1	
Season	September–March	254	52	20.5	0.091
	April–August	152	21	13.8	
Contact with cows	Yes	212	40	18.7	0.548
	No	192	32	16.6	
Contact with goats	Yes	283	59	20.9	0.014
	No	122	13	10.7	
Contact with sheep	Yes	50	8	16	0.809
	No	144	21	14.6	

### 3.2 Overall seroprevalence of *C. burnetii* infection

Table 1 summarizes the seroprevalence data of *C*. *burnetii* in the study subjects. Males had higher exposure (21.9%) than females (14.6%) but the difference did not reach statistical significance (*p* =0.056). Significant differences in seroprevalence were observed in different age categories, with individuals aged 6–12 years showing the highest seroprevalence (23.8%, *p* =0.002). Individuals who reported contact with goats had a higher exposure (20.9%) than those reporting no contact (10.7%, *p* =0.014). Contact with other animals did not increase the risk of exposure. Although not statistically significant, patients evaluated in the dry season had higher exposure (20.47%) compared to those evaluated in rainy season (13.82%, *p* =0.091).

### 3.3 Prevalence of acute and chronic Q fever

**FIGURE 1:**
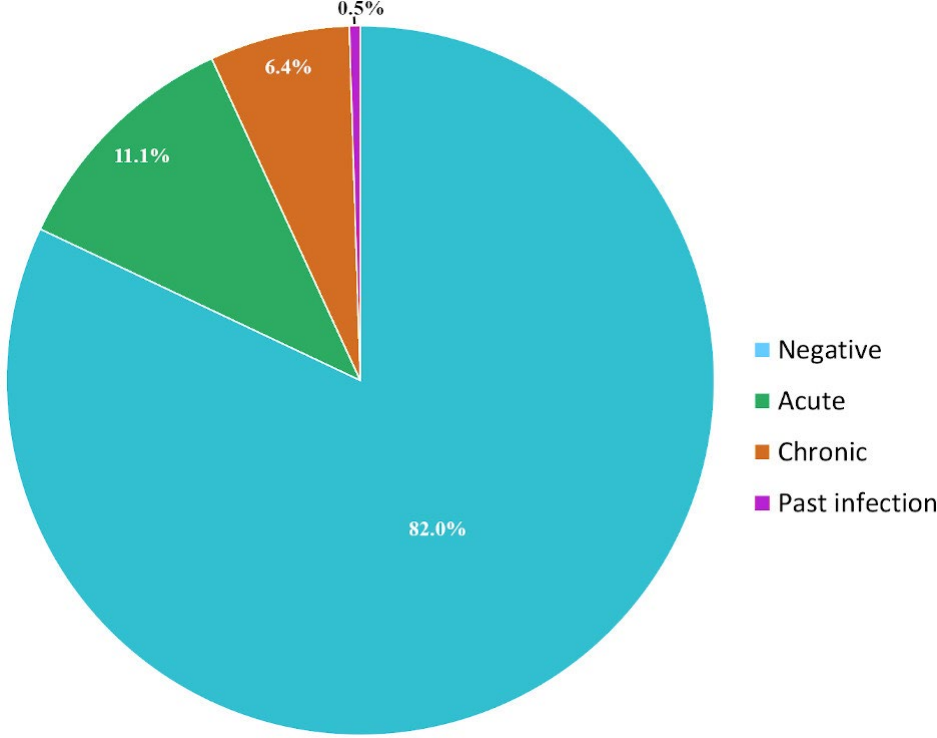
Q fever disease staged by immune responses to phase II and phase I antigens in patients attending Marigat Sub‐district with febrile illness

Seropositive samples were staged into acute if they had phase II IgM with or without IgG, or had IgG titers to phase II > IgG phase I, even in the absence of phase II IgM. Chronic infection if IgG to phase I titers was >800 or IgG to phase I > IgG to phase II. Past infection if IgG phase I titers was <800. Based on this, acute infections comprised 11.1% (45/406), 6.4% (26/406) for chronic and 0.5% (2/406) for past infection (Figure 1).

### 3.4 Q fever ELISA performance compared to IFA

We next assessed the performance of the Q fever ELISA for the detection of Phase II and Phase I immunoglobulins against IFA using a subset of samples (*n* = 93 for IgM and *n* = 100 for IgG antibodies), Table 2.

**TABLE 2:** *Coxiella burnetii* phase antigens by ELISA and IFA

		IFA IgM phase II (*n* = 93)			IFA IgG phase II (*n* = 100)			IFA IgG phase I (*n* = 100)		
		Positive	Negative	Total	Positive	Negative	Total	Positive	Negative	Total
ELISA Assay	Positive	8	8	16	36	0	36	37	1	38
	Negative	15	62	77	42	22	64	7	55	62
	Total	23	70	93	78	22	100	44	56	100

**FIGURE 2:**
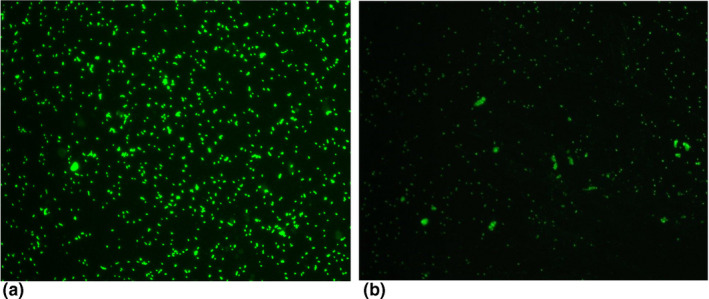
Typical photomicrographs for strong (panel A) and weak (panel B) IFA signals to phase II antigens of *Coxiella burnetii*. FITC labelled antibodies to phase II antigens show distinct apple green fluorescence of *C. burnetii* coco‐bacilli. Slides were observed at 400X magnification using an Olympus BX41 fluorescent microscope (Olympus America Inc. USA)

The agreement for detection of IgG phase I was 92% (92/100), with a kappa value of 0.84 (0.73–0.94) indicating a very good agreement rank. Crude agreement for IgG phase II and IgM phase II was much lower, 58.4% (59/100), kappa value of 0.28 (0.16–0.39) and 75.3% (70/93), kappa value of 0.26 (0.04–0.5), respectively. The calculated sensitivity for the Q fever ELISA IgM and IgG to phase II and IgG to phase I were 35%, 47% and 84% respectively with specificity of 89%, 100% and 97%, respectively. Figure 2 shows typical photomicrographs for strongly (panel A) and weak (panel B) IFA signals to phase II antigens of *C*. *burnetii*.

## 4 DISCUSSION

In Kenya, Q fever is rarely on the differential diagnosis radar by clinicians attending patients with febrile illness, despite the fact that a definite diagnosis can be made by serology. This is partly because the disease has very variable manifestation. The current study used immune responses to *C*. *burnetii* phase 1 and II antigens to infer presence of acute and chronic disease in patients presenting with febrile illness at Marigat District Hospital, Kenya. IFA is the gold standard for diagnosis but requires a fluorescent microscope that is rarely available in public hospitals. The utility of a commercial ELISA kit was evaluated against IFA on a subset of serum samples. Risk factors to *C*. *burnetii* infection were determined from the available demographic data.

The overall seroprevalence of Q fever in the population studied was 18.0%. This finding is consistent with previous studies conducted Kenya (Maina et al., 2016; Njeru et al., 2016). In studies targeting patients with acute lower respiratory illness, a common presenting sign of Q fever, a seroprevalence of 30.9% has been reported (Knobel et al., 2013). Community seroprevalence is however lower, with one study reporting 2.5% (Wardrop et al., 2016). One of the drivers of Q fever is presence of the bacteria in animals. Muema et al. (2017) reported herd seroprevalence of 12.2% and 26.0% in sheep and goats, respectively (Muema et al., 2017). Another study reported a higher seroprevalence in sheep (57%) and goats (83.1%) that were going through abortions (Nakeel et al., 2016). The major economic activity of our study population is nomadic pastoralism and agro‐pastoralism who keep large herds of ruminants. The seroprevalnce of *C*. *burnetii* observed in the pastoralists attending hospital with acute febrile illness is attributable to the close interaction with infected animals.

As shown in Figure 1, 18% of the study patients had antibody signatures to Q fever, of which 11% were for acute disease, 2% chronic and 6% for previous exposure. Clearly, Q fever is a significant morbidity to pastoral community in Marigat. Q fever is not restricted to Marigat. Recent studies indicate similar morbidity estimates in Western Kenya (Maina et al., 2016) and North Eastern Kenya (Njeru et al., 2016). A 5% seroprevalence of acute Q fever has also been reported in neighbouring Tanzania (Prabhu et al., 2011) and 9.5% in Mali, West Africa (Steinmann et al., 2005).

Our result indicated that gender, age, season and animal contact as the main risk factors for *C*. *burnetii* seropositivity. As shown in Table 1, more men (21.9%) were exposed to the infection compared to women (14.6%, *p* =0.056). This finding may be attributed to the fact that in traditional pastoral communities, it is the men who are in close association with animals when performing chores such as herding, milking and assisting in removal of retained placenta or handling aborted fetuses. As was reported previously, men herders interact closely and for longer periods with animals compared to the female counterparts (Muga et al., 2015). Similar gender bias in Q fever predisposition has been reported in other studies (Schelling et al., 2003). In mice, it has been shown that, estradiol, the predominant female sex hormone is protective against intracellular bacterial infections (Leone et al., 2004).

In our study, the most affected age group was between 6 and 12 years with a prevalence rate of 23.8% (Table 1). The occurrence of Q fever in children has been reported globally (Kobbe et al., 2008). This is dissimilar to previous studies that showed greater risk in older persons. For example, Njeru et al. (2016) noted that older persons (25–60 years) faced a greater risk of infection due to cumulative risk of exposure and adult male dominance in risky occupations than children. Another study by Nakeel et al. (2016) found higher seroprevalence in adults (34%) compared to adolescents and children (23% and 26% respectively). Immunological naiveté of children has been reported as a predisposing factor (26). Our result illustrates that Q fever is not just an occupational hazard among adults but also afflicts children.

Although not statistically significant, seropositivity was highest (20.5%) in the dry season (Sep–Mar) compared to 13.8% in the rainy season (April–August; Table 1). Seasonal trends in Q fever have been reported in other countries (Gardon et al., 2011; Maurin & Raoult, 1999). In Japan, most of the Q fever cases are reported in winter. On the other hand, in Germany most of the cases were reported in summer (Hellenbrand et al., 2001) and in autumn in Cyprus (Cantas et al., 2011). It is probable that dry environment favours transmission of aerosolized *C*. *burnetii* spores.

Contact with goats was a strong risk for exposure (Table 1), but not with other animals. In a recent outbreak in Netherlands, keeping large number of goats was associated with the occurrence of Q fever in humans and people living closer to large farms with over 800 goats were at a higher risk of contracting *C*. *burnetii* infection (Dijkstra et al., 2012).

The gold standard for Q fever diagnosis is IFA. We therefore assessed the performance of the commercial Q fever ELISA test kit (Table 2). ELISA was only useful in detection of IgG to phase I antigens (chronic Q fever) with a kappa value 0.84 (0.73–0.94). Crude agreement for IgG phase II and IgM phase II was much lower at kappa value of 0.28 (0.16–0.39) for IgG and 0.26 (0.04–0.5) for IgM. Similar results have been reported before (Kantsø et al., 2012). Surprisingly, Meekelenkamp et al. (2012) found a very high sensitivity (85.7%), and specificity (97.6%) for phase II IgM (Meekelenkamp et al., 2012). This could be explained by the fact that they tested serum 2 weeks after the onset of symptoms and during a period of extremely high incidence (outbreak), a time when serum IgM antibodies to phase II are at their peak concentration.

In conclusion, the Q fever ELISA used in this study underestimated acute Q fever by nearly 50% (sensitivity of 46%). It is therefore likely that the true prevalence of IgM to phase II antigens in the febrile patients seeking treatment at Marigat District Hospital is double the estimate by ELISA. Compared to phase II IgM, the seroprevalence for phase I IgG was low, despite high sensitivity and specificity of the test method. Therefore, a community‐based study will be needed in order to inform the true seroprevalence of Q fever in the Marigat population.

## DISCLAIMER

Material has been reviewed by the Walter Reed Army Institute of Research. There is no objection to its presentation and/or publication. The opinions or assertions contained herein are the private views of the author and are not to be construed as official, or as reflecting true views of the Department of the Army or the Department of Defense. The investigators have adhered to the policies for protection of human subjects as prescribed in AR 70–25.

## CONFLICT OF INTEREST

No competing financial interests exist.

## AUTHOR CONTRIBUTION

**Allan P Lemtudo:** Data curation; Formal analysis; Investigation; Methodology; Writing‐original draft. **Beth K Mutai:** Investigation; Project administration; Writing‐review & editing. **Lizzy Mwamburi:** Supervision; Writing‐original draft; Writing‐review & editing. **John Waitumbi:** Conceptualization; Formal analysis; Funding acquisition; Investigation; Methodology; Project administration; Resources; Supervision; Writing‐review & editing.

### PEER REVIEW

The peer review history for this article is available at https://publons.com/publon/10.1002/vms3.493.

## Data Availability

The data that support the findings of this study are available from the corresponding author upon reasonable request.
